# Aristolochic Acid-Induced Genotoxicity and Toxicogenomic Changes in Rodents

**DOI:** 10.4103/wjtcm.wjtcm_33_19

**Published:** 2020-03-13

**Authors:** Xi-Lin Li, Xiao-Qing Guo, Hai-Rong Wang, Tao Chen, Nan Mei

**Affiliations:** a Division of Genetic and Molecular Toxicology, National Center for Toxicological Research, Jefferson, AR, USA,; b Tianjin Center for New Drug Safety Assessment and Research, Tianjin, China

**Keywords:** Aristolochic acid, benchmark dose, genotoxicity, mutation, toxicogenomics

## Abstract

Aristolochic acid (AA) is a group of structurally related nitrophenanthrene carboxylic acids found in many plants that are widely used by many cultures as traditional herbal medicines. AA is a causative agent for Chinese herbs nephropathy, a term replaced later by AA nephropathy. Evidence indicates that AA is nephrotoxic, genotoxic, and carcinogenic in humans; and it also induces tumors in the forestomach, kidney, renal pelvis, urinary bladder, and lung of rats and mice. Therefore, plants containing AA have been classified as carcinogenic to humans (Group 1) by the International Agency for Research on Cancer. In our laboratories, we have conducted a series of genotoxicity and toxicogenomic studies in the rats exposed to AA of 0.1–10 mg/kg for 12 weeks. Our results demonstrated that AA treatments induced DNA adducts and mutations in the kidney, liver, and spleen of rats, as well as significant alteration of gene expression in both its target and nontarget tissues. AA treatments altered mutagenesis- or carcinogenesis-related microRNA expression in rat kidney and resulted in significant changes in protein expression profiling. We also applied benchmark dose (BMD) modeling to the 3-month AA-induced genotoxicity data. The obtained BMDL_10_ (the lower 95% confidence interval of the BMD_10_ that is a 10% increase over the background level) for AA-induced mutations in the kidney of rats was about 7 μg/kg body weight per day. This review constitutes an overview of our investigations on AA-induced genotoxicity and toxicogenomic changes including gene expression, microRNA expression, and proteomics; and presents updated information focused on AA-induced genotoxicity in rodents.

## Introduction

Aristolochic acid (AA) refers to a group of structurally related nitrophenanthrene carboxylic acids (individual or mixture) found in plants of the Aristolochiaceae family, which is comprised of more than 450 species primarily in the *Aristolochia* and *Asarum* genera.^[1–3]^ There are two major forms of AA, 8-methoxy-6-nitronaphtho[2,1-g][1,3] benzodioxole-5-carboxylic acid, and 6-nitronaphtho[2,1-g] [1,3]benzodioxole-5-carboxylic acid, according to the International Union of Pure and Applied Chemistry, which are commonly referred to as AAI (CAS# 313–67-7) and AAII (CAS# 475–80-9), respectively.^[4]^ Since antiquity, herbal remedies containing AA have been widely used in African, Asian, European, and South American countries for medicinal purposes, such as for the treatments of snakebite, edema, and inflammatory diseases.^[2,5]^ In traditional Chinese medicines, the *Aristolochia* species (such as Ma Dou Ling, Mu Tong, Guang Fang Ji, and Tian Xian Teng) were prescribed for hepatitis, headache, upper respiratory tract infection, heart diseases, and many other conditions.^[6]^

As early as 1825, the toxicity of certain *Aristolochia* species (e.g., *Aristolochia serpentaria*) in humans was reported, and in the 1950s, toxicity associated with *Aristolochia clematitis* in horses was observed.^[7,8]^ In the 1980s, Mengs *et al.* reported that subchronical or chronical treatments of rats and mice with AA through gavage resulted in tumors in multiple tissues. When rats were gavaged with AA at the doses of 0.1, 1, and 10 mg/kg body weight (bw) for 3 months, the incidence of tumors in the forestomach, kidney, renal pelvis, and urinary bladder was about 25%, 85%, and 100% at these three doses, respectively.^[9]^ Within 1 year after the mice were treated with 5 mg/kg AA for 3 weeks, carcinomas in the forestomach, glandular stomach, kidney, lung, and uteri were observed.^[10]^ The toxicity and carcinogenicity of AA in humans first caught attention in the early 1990s. A group of Belgian women who consumed AA-containing herbal products including *Aristolochia fangchi* (Fang Ji) as part of a weight-loss regimen developed progressive interstitial renal fibrosis.^[11,12]^ Urothelial cancer was identified in about half of these patients within a decade, and urothelial dysplasia was found in 19 of 21 patients without cancer.^[13]^ Since then, similar cases of AA-associated renal fibrosis and upper urinary tract tumor formation were reported in other parts of the world.^[14,15]^ Given its unique pathological and morphological features, the term “Chinese herbs nephropathy,” later replaced by a more accurate name “Aristolochic acid nephropathy” (AAN), was used to describe this “new” type of renal failure induced by AA.^[16,17]^ It is worth noting that a high number of Balkan endemic nephropathy (BEN) cases, first described in the late 1950s, have been linked to AAN cases.^[18,19]^ These findings suggest that the carcinogenic potential of AA was underestimated for a period of time.

Given evidence of AA’s toxicity and carcinogenicity in humans and in experimental animals, International Agency for Research on Cancer (IARC) classified products containing AA as Group 2A human carcinogen in 2002 (IARC 2002). Based on new data, a few years later, IARC reclassified AA as a Group 1 human carcinogen.^[20]^ Therefore, AA-containing products have been banned by many regulatory agencies in most countries. In 2001, the United States Food and Drug Administration (U. S. FDA) issued a warning regarding the potential cancer risks of AA contained in some dietary supplements or botanical products,^[21]^ and in 2011 and 2019, the FDA maintained an import alert that prohibited AA-containing dietary supplement products from entering the U. S. market.^[22]^ Despite these efforts, traditional medicines containing AA are still marketed worldwide. For example, such products were/are available on the internet.^[23]^ Using a liquid chromatography–mass spectrometry approach, 25 of 190 Chinese traditional herbal preparations from the Dutch market had AA contamination.^[24]^ Similarly, AAI and AAII were present in 20% and 7% of 30 herbal products purchasable in the U. S. through the Internet.^[25]^ Between 1997 and 2003, it was estimated that one-third of the population in Taiwan could potentially be exposed to AA through the prescription of traditional Chinese medicine, which may contribute to the high incidence of kidney failure and upper urinary tract tumor.^[26,27]^ AAN still occurs occasionally in Korea, where AA has been prohibited since 2005, probably because of the availability of AA-containing drugs in oriental clinics.^[28]^ Therefore, the human exposure to AA still exists widely and may be underestimated due to the complexity of the nomenclature of traditional Chinese medicines.

AA (either individual AA or mixtures of AA) was nominated by the National Institute of Environmental Health Sciences (located at Research Triangle Park, NC, USA) to the U. S. National Toxicology Program (NTP) for possible listing in the Report on Carcinogens, and in 2008, the NTP released its final report on AA.^[1]^ During the last two decades, the toxic, genotoxic, and carcinogenic properties of AA have been studied in *in vivo* and *in vitro* experimental models,^[29,30]^ and the data from human exposure and cancer cases, and experimental animals have been reviewed.^[16,31–34]^ In our laboratories, we investigated DNA damage and mutations in the *cII* gene of target and nontarget organs of male Big Blue transgenic rats gavaged with AA (0.1–1 mg/kg bw) for 3 months.^[35–37]^ We determined a significant dose-dependent induction of H-*Ras* mutant fraction of codon 61 CAA → CTA mutation in the kidney and liver.^[38]^ We observed that more differentially expressed genes (DEGs) involved in cancer-related pathways occur in the kidney rather than in the liver of exposed rats using microarray analysis^[39]^ and identified tissue-specific microRNA responses in AA-treated rats.^[40]^ A review article focused on *in vivo* and *in vitro* genotoxicity of AA was published in 2007.^[32]^ In the past decade, efforts have focused on assessing the potential mechanisms of AA-induced carcinogenesis, using *in vivo* models and toxicogenomic technologies. In the current review, we summarize these studies in rodents (mice and rats) and discuss the possible mechanisms underlying the genotoxicity and carcinogenicity of AA.

## Metabolism of Aristolochic Acid *In Vivo*

**Figure 1:**
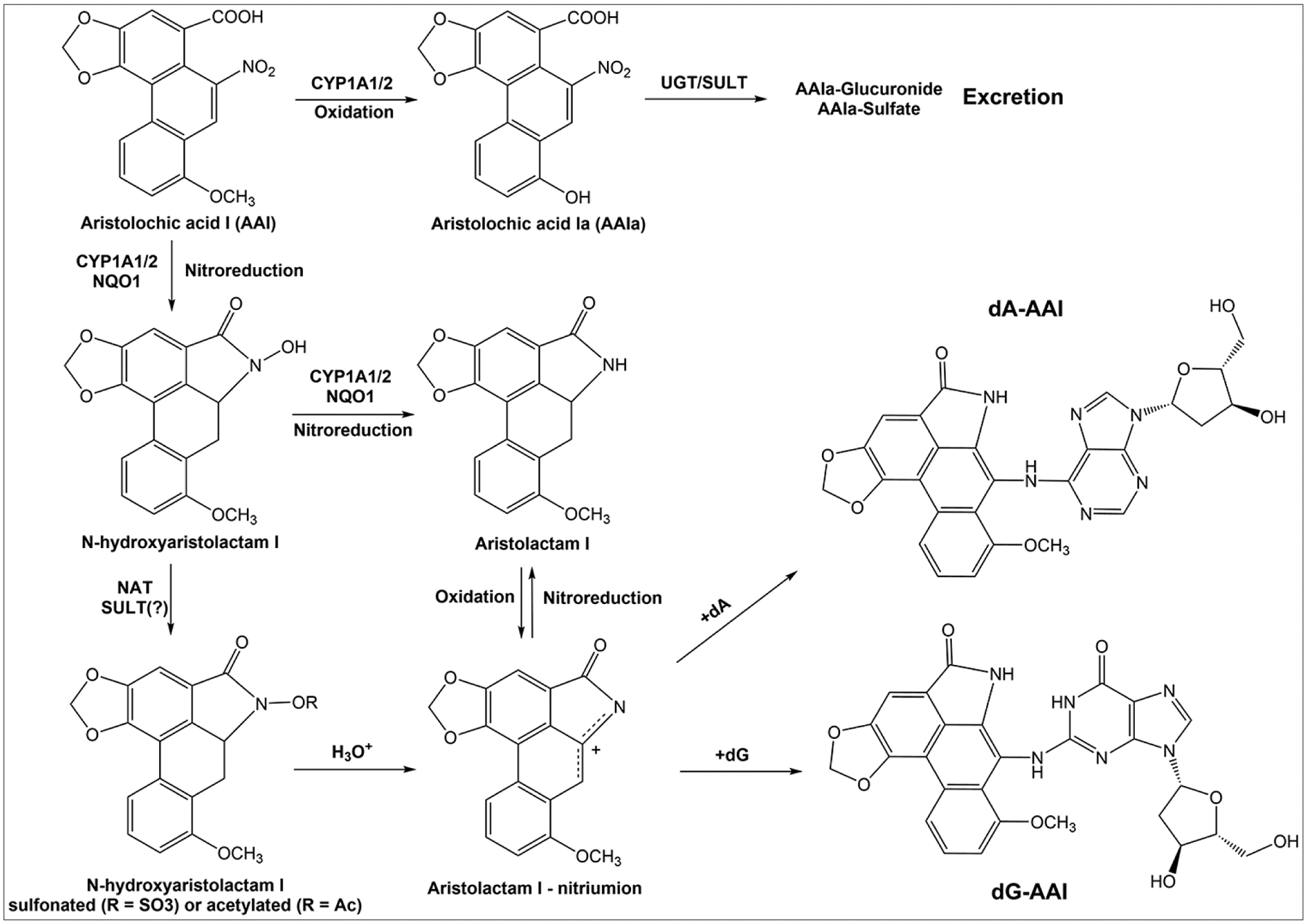
Bioactivation and detoxification of aristolochic acid I by metabolic enzymes. UGT: uridine 5’-diphospho-glucuronosyltransferase, SULT: sulfotransferase, NAT: N-acetyltransferase. The question mark (?) indicates that the role of sulfotransferase in the bioactivation of aristolochic acid is controversial

Metabolic activation is often required for procarcinogens to become carcinogens *in vivo*. AAI and AAII, the major two components of AA, can be metabolized to their corresponding aristolactams under the anaerobic conditions by phase I metabolic enzymes.^[41]^ While aristolactams are generally not genotoxic, aristolactam–nitrenium ions, the intermediate product generated during the metabolic procedure, can directly react with DNA and give rise to deoxyguanosine and deoxyadenosine adducts. Nitroreduction is the key step for the biotransformation of AA to reactive nitrenium ions. In the past several years, multiple enzymes have been studied using rodent models to examine their potential roles in nitroreduction reactions and the bioactivation of AA [Figure 1].

NAD(P)H:quinone oxidoreductase 1 (NQO1), a cytosolic reductase that is highly expressed in the kidney, has been implicated in mouse AAN.^[42]^ The inhibition of NQO1 enzymatic activity by pretreatment with dicoumarol and phenindione (NQO inhibitors) attenuated AAI-induced nephrotoxicity in C57BL/6 mice. When NQO1 activity was suppressed, the formation of aristolactam I was significantly reduced along with an increase in levels of 8-OH-AAI (AAIa), a much less toxic metabolite of AAI. In mice, the protein level and enzymatic activity of NQO1 in both liver and kidney were significantly elevated by the administration of AAI at a dose of 50 mg/kg bw.^[43]^ This result not only supports the role of NQO1 in AA bioactivation but also indicates that AAI may enhance its own genotoxicity through the induction of NQO1.

Phase I enzymes, particularly CYP1A1/2, can lead to both activation and detoxification of AAI in rodents.^[44]^ Under aerobic conditions, AAI can be detoxified to AAIa by hepatic CYP1A1/2 through demethylation. AAIa appears to be minimally genotoxic. A previous *in vivo* study showed that the DNA adducts derived from AAIa represented only 1% of these derived from AAI in the renal cortex.^[45]^ Pretreatment with β-naphthoflavone, a commonly used aryl hydrocarbon receptor agonist (CYP1A inducer), facilitated the clearance of AAI and thereby reduced the formation of AAI-DNA adducts.^[46]^ This oxidative detoxification pathway can attenuate AA-associated renal failure in mice.^[47]^ However, under anaerobic conditions, both AAI and AAII undergo reductive activation by CYP1A enzymes and form reactive nitrenium ions, which can bind directly to DNAs to form adducts. Therefore, oxygen concentration seems to be crucial in AA metabolism and has been suggested to contribute to AA-induced tissue-specific genotoxicity.^[44]^ In addition, a recent study suggested that Phase II enzymes such as sulfotransferases and N-acetyltransferases appear to further increase the genotoxicity of AA by converting N-hydroxyaristolactam I to its sulfonated and acetylated conjugates, which then facilitate the formation of reactive nitrenium ions.^[48]^ Nevertheless, another study did not find an increased formation of AAI- and AAII-DNA adducts in mice expressing human SULT1A1/1A2, suggesting that sulfotransferases may not be crucial in the bioactivation of AA *in vivo*.^[49]^ Further studies are needed to confirm the roles of sulfotransferases and *N*-acetyltransferases in the metabolic activation of AA.

## Aristolochic Acid-Induced DNA Damage

### DNA adduct formation

The 7-(deoxyadenosin-*N*^6^-yl)-aristolactam I (dA-AAI) is the major DNA adduct found in multiple tissues of humans exposed to AA-containing products.^[50,51]^ In fact, dA-AAI has been proposed to be a reliable biomarker of AA consumption since it can be detected in patients’ renal tissues after decades of exposures.^[52]^ In rodents, AA-induced tumor formation only appears in the kidney, forestomach, and urinary tract. Therefore, an initial study was conducted to survey the tissue-wide levels of AA-derived DNA adducts. Male Wistar rats were treated with AAI and AAII at an oral dose of 10 mg/kg/day for 5 days, and the DNA adduct levels in different tissues were evaluated using the ^32^P-postlabeling method.^[53]^ While the highest level of dA-AAI was showed in the forestomach (about 330 per 10^8^ nucleotides) and a relatively low level in the kidney, dA-AAI was also found in other nontarget tissues including glandular stomach, liver, stomach, and urinary bladder epithelium. Although at lower levels, dA-AAII was detected in these tissues as well. Subsequently, two other studies from the same research group were carried out to examine the persistence of DNA adducts in several organs of rats that were administered a single oral dose of AAI.^[54,55]^ These animals were sacrificed at various times after treatment (up to 36 weeks). The results showed both dA-AAI and 7-(deoxyguanosin-*N*^2^-yl)-aristolactam I (dG-AAI) were rapidly removed from the tissues (kidney, liver, lung, stomach, forestomach, and urinary bladder), with both DNA adducts reducing to about 50% in the kidney after 1 day of treatment. However, while dG-AAI was quickly diminished, about 25% of the dA-AAI remained in the tissues between 4 and 36 weeks.

**Figure 2:**
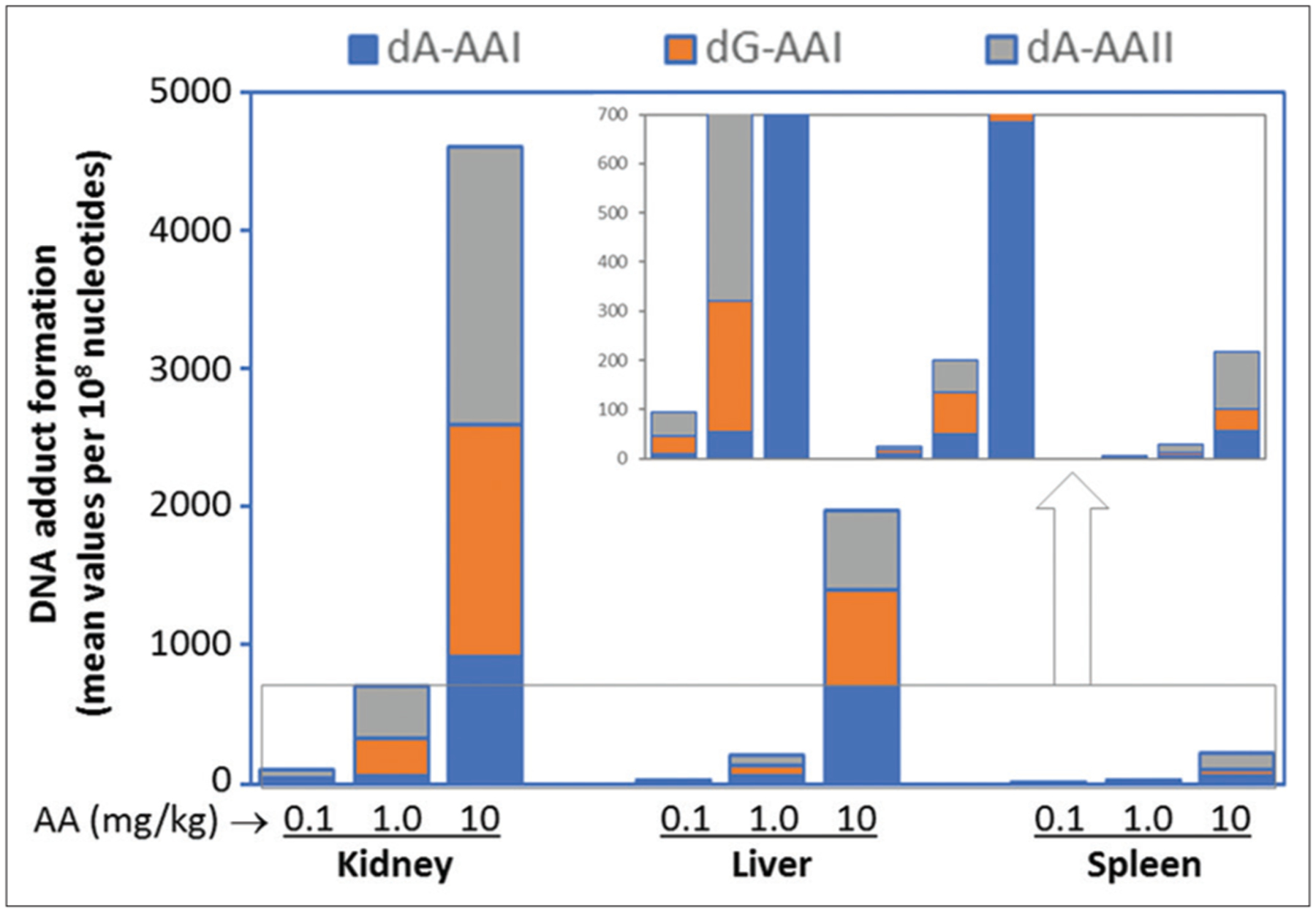
Total DNA adduct levels in the kidney, liver, and spleen of Big Blue rats treated with aristolochic acid at different doses (0.1, 1.0, and 10.0 mg/kg body weight) for 12 weeks. All DNA adducts (dA-AAI, dG-AAI, and dA-AAII) were detected by the nuclease P1 enrichment version of the ^32^P-postlabeling assay. The kidney and liver data are from Mei *et al*.^[35]^ while the spleen data are from McDaniel *et al*.^[37]^ All data represent the mean value of groups of 6 rats. The part of DNA adducts under 700/10^8^ nucleotides has been enlarged in the inset graph

In our laboratory, we compared the DNA adduct formation in the kidney, liver, and spleen of Big Blue transgenic rats treated with AA (containing 40% AAI and 56% AAII) at the doses of 0.1, 1, and 10 mg/kg for 3 months.^[35,37]^ The selected doses and treatment schedule were matched to a previous carcinogenicity study in rats.^[9]^ A subchronic repeated dose study can more closely approximate human exposure pattern than single-dose administration. Three major DNA adducts (dA-AAI, dG-AAI, and dA-AAII) were induced in the kidney, liver, and spleen in a dose-dependent manner, as measured by the ^32^P-postlabeling assay [Figure 2]. The kidney had the highest DNA adduct level among the three tissues (kidney vs. spleen, >20-fold; liver vs. spleen, 5–9-fold). The amount of dA-AAI in the liver and kidney was similar at all doses studied. Interestingly, the kidney (the target tissue) appeared to have more dG-AAI and dA-AAII adducts than the liver. At the carcinogenic dose of 10 mg/kg bw, dG-AAI and dA-AAII in the kidney were 2.3-and 3.6-fold higher than those in the liver, respectively.^[35]^ In the spleen, dA-AAII seemed to be the major DNA adduct, and the level of dA-AAII was > 2-fold higher than dA-AAI and dG-AAI at all doses studied.^[37]^ Whether the higher levels of dG-AAI and dA-AAII in the kidney eventually contribute to the tumor formation is still unclear. It should be noted that the tissues were harvested 1 day after the last dosing in these studies. As mentioned previously, dA-AAI is the major DNA adduct found in humans and its persistence has been proposed to be directly correlated with renal malignancy. To better interpret the role of dG-AAI and dA-AAII in renal cell carcinoma, future studies are required to examine their temporal occurrence across tissues during the subchronic exposure.

More recently, using the liquid chromatography with tandem mass spectrometry (LC-MS/MS) technique, dA-AAI and dG-AAI were identified in Sprague-Dawley rats given a single AAI dose of 10 or 30 mg/kg bw.^[56]^ Similar to the previous studies using ^32^P-postlabeling, the highest amount of dA-AAI was found in the forestomach. In addition to the target tissues, dA-AAI was detected in the heart, small intestine, and large intestine 1 day after the dosing. A time-course kinetic analysis of the DNA adducts indicated that the average half-lives of dA-AAI were about 19.7 and 2.8 days in the target tissues (forestomach and kidney), which further explained the persistence of dA-AAI in animals. Using LC-MS/MS, the same research group also reported that the formation of AA-RNA adducts was significantly greater than AA-DNA adducts in both liver and kidney of rats.^[57]^ The linkage between AA-RNA adducts and its carcinogenicity, however, remains unknown.

Chemical interactions may have further influence on the AA-DNA adduct formation. For example, ochratoxin A is a renal toxic food contaminant and that was proposed to be linked with the development of BEN.^[58]^ Co-exposure to AA (about 33% AAI and 64% AAII) and ochratoxin A resulted in a significant increase of AA-DNA adduct formation in both liver and kidney in rats.^[59]^ The suppression of detoxification pathways by ochratoxin A appears to account for the enhancement of AA’s genotoxicity.

### Comet assay

The *in vivo* comet assay is often conducted to measure chemical-induced DNA damage and strand breaks in a target tissue. AA-induced DNA damage was examined using the comet assay in several *in vivo* studies. In isolated renal cells from male Sprague-Dawley rats treated with a single AA dose (20 or 40 mg/kg, containing 27% AAI and 65% AAII), the median Olive tail moment of DNA in the treated groups was significantly elevated 22–26 h after the treatments.^[60]^ Sampling time appeared to play an important role in AA-induced DNA damage, as the increased Olive tail moment was not identified after 3–6 h of dosing. In another study, DNA double-strand breaks were detected by the alkaline comet assay in the bone marrow, liver, and kidney cells of F344 rats administered with 11, 22, and 30 mg/kg AA (1:1 mixture of AAI and AAII) for 4 consecutive days.^[61]^ The magnitude of the AA-induced DNA damage, as measured by percentage of DNA in tail (% tail DNA), was similar across all tissues studied. No apparent dose-responsive pattern in the increased % tail DNA was observed, which could be partially due to a narrow dose range and sampling time window that were used in this study. Histopathological examination was performed to evaluate the potential cytotoxicity of AA in the kidney and liver. Given that only moderate nephrotoxicity was seen in AA-treated rats, it was unlikely that the observed DNA damages were caused by cytotoxicity, at least in the liver.

### Oxidative DNA damage

Overproduction of reactive oxygen species (ROS) may lead to oxidative DNA damage that initiates and promotes chemical carcinogenesis.^[62]^ The induction of oxidative DNA damage (as measured by the formation of 8-hydroxyguanosine) and depletion of glutathione by AA have been demonstrated using *in vitro* models.^[63,64]^ In mice and rats, AA attenuated the overall antioxidant capacity in the kidney. A reduced level of glutathione accompanied by an impaired intrarenal antioxidant capacity was found in the kidney of mice treated with 10 mg/kg AA for 5 days.^[65]^ Concordantly, the gene expression of ROS-generating enzymes such as *Nox2* was upregulated in the plasma of male C57BL/6 mice exposed to a low-dose of AA.^[66]^ The supplementation of l-arginine, a nitric oxide precursor with antioxidant effects, significantly decreased *Nox2* expression and ROS production, and improved renal functions in mice treated with AA. This evidence clearly supports the conclusion that AA can induce oxidative stress, especially in renal tissues. However, no study has been systematically conducted to investigate whether AA can induce oxidative DNA damage in rodent models and whether oxidative stress can be a potential mechanism for AA’s carcinogenicity.

### Micronucleus assay

The *in vivo* micronucleus test using bone marrow cells or peripheral blood cells is a critical tool to assess the genotoxicity of chemicals. Overall, the micronucleus response to AA exposure is not strong in rodents and appears to be impacted by treatment regimens. A previous study showed that a number of micronucleated polychromatic erythrocytes were significantly greater in both male and female mice treated with AA (77% AAI and 21% AAII) for 48 h than in the concurrent vehicle controls.^[67]^ In that study, animals were dosed through a single intravenous injection, and the doses of AA ranged from 6 to 60 mg/kg. However, in another study using lambda/lac*Z* transgenic mice exposed to 15 mg/kg AA (56% AAI and 44% AAII) once a week for 4 weeks, the frequency of micronucleated reticulocytes (RETs) in the peripheral blood cells was not changed.^[68]^ In rats, AA induced a minimum micronucleus response. The percentage of micronucleated RETs in peripheral blood increased slightly in F344 rats treated with 11 mg/kg AA (1:1 mixture of AAI and AAII) for 3 days as compared to controls but did not increase using lower doses.^[61]^ On day 29 after the AA administration, no induction of micronucleated RETs was observed in any dose group. An immediate follow-up experiment attempting to examine the clastogenic effects of AA at higher doses (22 and 30 mg/kg/day) showed negative results in the micronucleus tests. Taken together, the current weight of evidence suggests that AA is a weak clastogen *in vivo*.

## Aristolochic Acid-Induced Mutations in Gene Mutation Assays

### *cII* transgene

Transgenic mutation assays have been used to detect gene mutations in multiple organs of transgenic animals (rats and mice). The Muta Mouse and Big Blue transgenic rodents are the most widely used screening systems for mutagens *in vivo*. The *cII* gene is a sensitive and efficient reporter of mutation in both assays.^[69]^ Using the Muta Mouse, treatment with a single dose of 15 mg/kg AA once a week for 4 weeks increased the *cII* mutant frequency (MF) 15-, 9-, and 31-fold over the controls in the forestomach, kidney, and bladder, respectively.^[68]^ The result from sequencing analysis revealed that AA caused a signature A:T to T:A transversion in the target tissues. On the other hand, *cII* MF was not significantly increased in the nontarget tissues, such as liver, spleen, and lung. Interestingly, the *cII* MF was increased about 9-fold in the colon, which was recognized recently as a possible AA target.^[70]^

**Figure 3:**
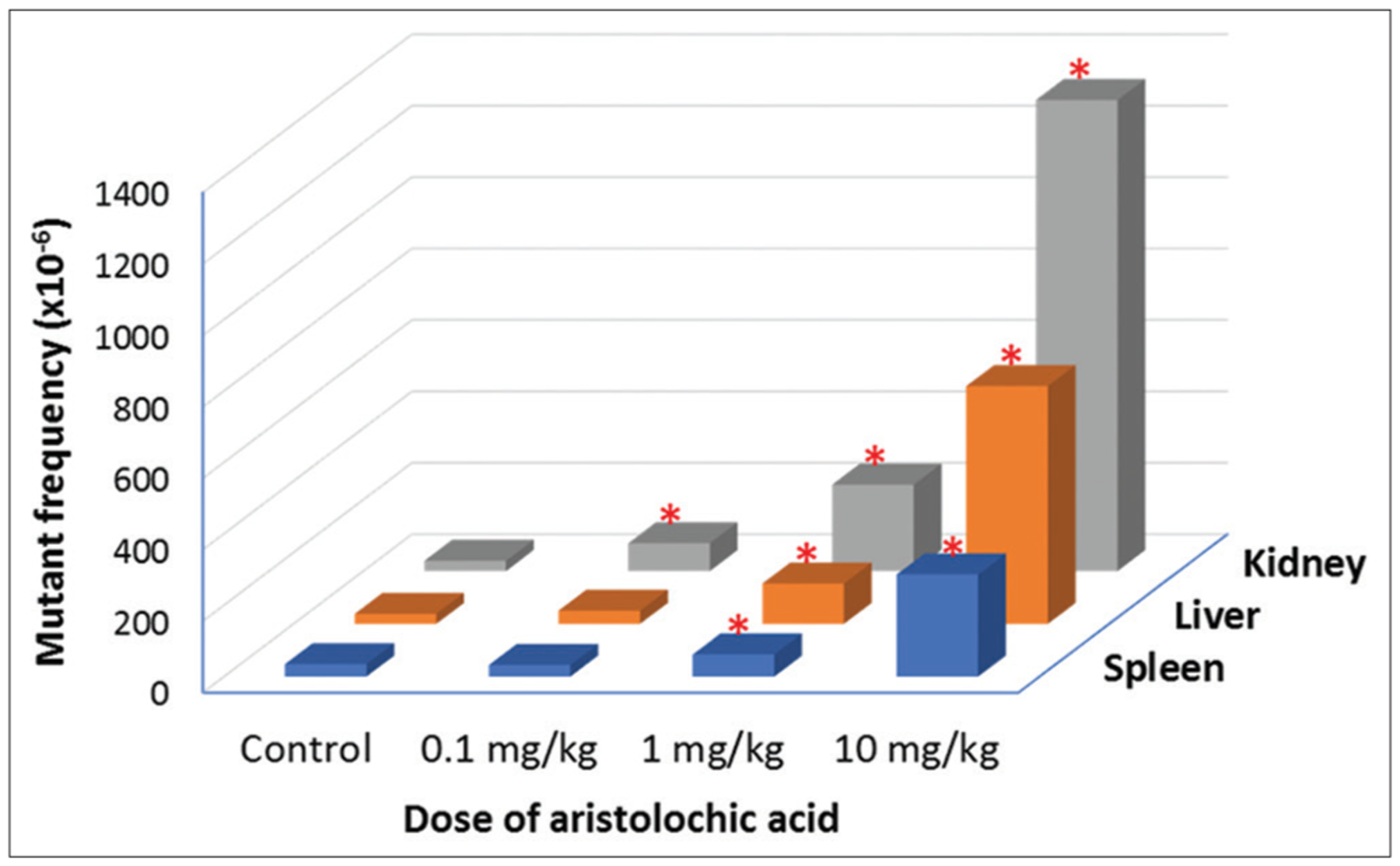
The *cII* mutant frequencies in the kidney, liver, and spleen of Big Blue rats treated with aristolochic acid at different doses (0.1, 1.0, and 10.0 mg/kg body weight) for 12 weeks. The data represent the mean value for groups of 6 rats. The kidney data are from Chen *et al*.,^[36]^ while the liver and spleen data are from Mei *et al.*^[35]^ and McDaniel *et al*.,^[37]^ respectively. *Indicating a significant difference from the concurrent control group

**Figure 4:**
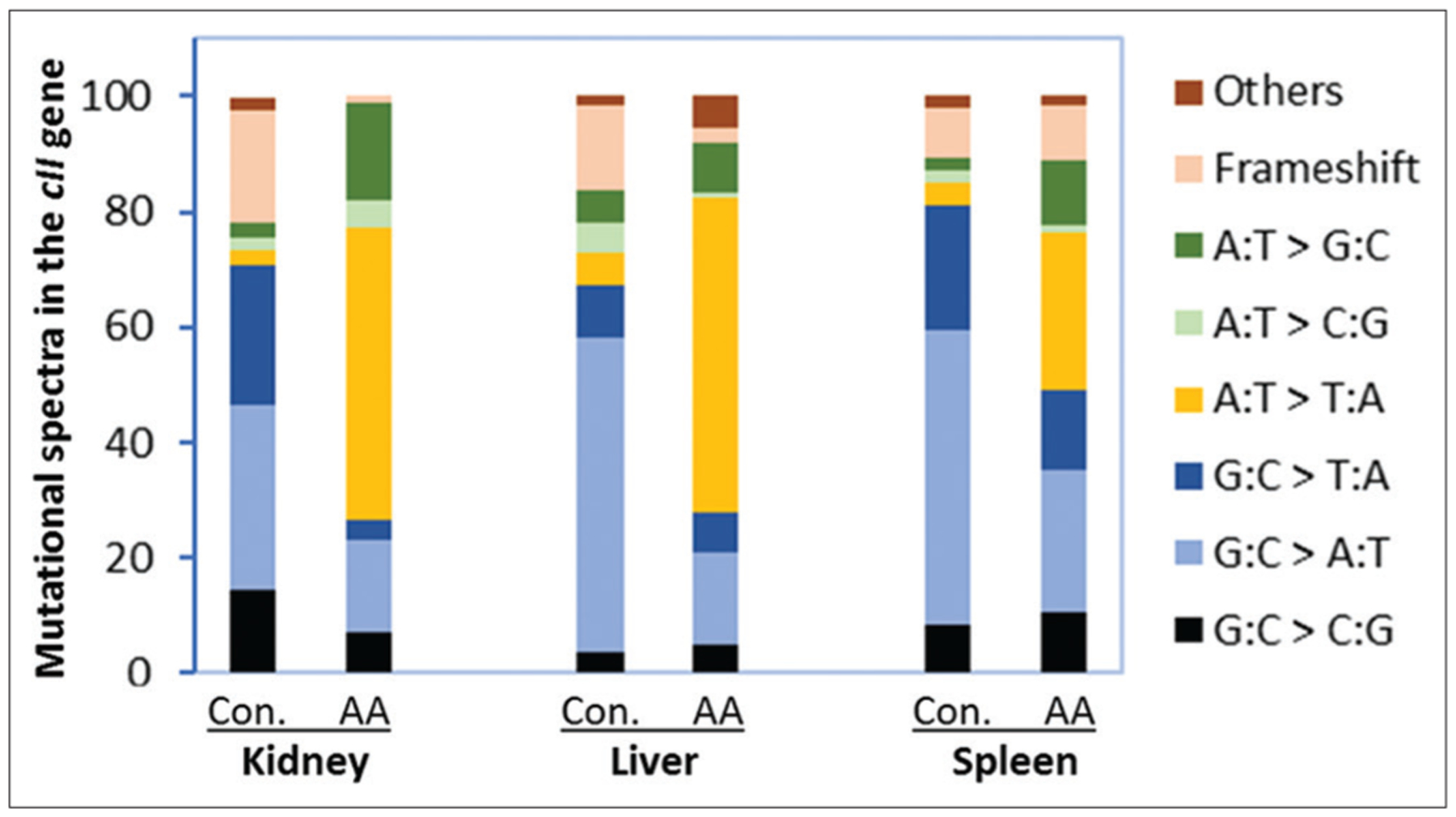
The summary of independent mutations in the *cII* gene of the kidney, liver, and spleen from control and 10 mg/kg aristolochic acid-treated rats. The kidney data are from Chen *et al.*,^[36]^ while the liver and spleen data are from Mei *et al.*^[35]^ and McDaniel *et al.*,^[37]^ respectively. There are statistically significant differences between the mutation spectra in the control and AA-treated rats for each tissue. The group of “others” includes tandem base substitution and complex mutation

We also investigated the mutagenicity of AA in Big Blue transgenic rats.^[35–37]^ AA was administered to the rats through oral gavage at doses ranged from 0.1 to 10 mg/kg for 12 weeks. The Select-*cII* Mutation Detection System was used to identify the MF in the kidney, liver, and spleen. We found that AA increased the *cII* MF in a linear dose-responsive manner in all organs studied. The magnitude of MF ranged from 29 to 1319 × 10 ^− 6^, 28–666 × 10 ^− 6^, and 35–286 × 10 ^− 6^ in the kidney, liver, and spleen, respectively [Figure 3]. As with the Muta Mouse model, A:T to T:A transversion was the predominant mutation type induced by AA while G: C to A:T transition appeared to be the major type of spontaneous mutations [Figure 4]. A:T to T:A transversion accounts for 54%, 50%, and 27% of all independent mutations in the liver, kidney, and spleen of rats exposed 10 mg/kg, respectively. In contrast, the A:T to T:A transversion in controls was below 5% in all the tissues studied. It is worth noting that in the spleen study, we sequenced all mutants from low-, middle-, and high-dose groups, and the percentages of A:T to T:A mutations increased dose dependently to 10%, 17%, and 27%, respectively.^[37]^ These findings indicate that mutagenicity, particularly A:T to T:A transversion, is a key mode of action (MOA) for the tumor induction by AA.

### *lacZ* transgene

As a reporter gene for mutation, the *lacZ* transgene was incorporated into the genome of Muta Mouse within the lambda phage vector, and the *lacZ* MF can be determined by *lacZ*-positive selection of DNA samples recovered from tissues of interest. It is one of the transgenic rodent mutation assays that can detect tissue-specific mutations. Accordingly, the *lacZ* gene in Muta Mouse was used to evaluate the mutagenicity of AA in multiple tissues and organs. The mice were exposed to either vehicle or a weekly dose of 15 mg/kg AA for 4 weeks.^[68]^ In AA target organs, including kidney, forestomach, and bladder, the *lacZ* MFs increased 10-, 33-, and 16-fold in the AA-treated groups over their concurrent controls. As expected, AA did not induce significantly higher MFs in the *LacZ* gene in the nontarget organs, except for colon. Notably, despite the close anatomical position, forestomach (1129 ± 262 × 10 ^−6^) and granular stomach (141 ± 29 × 10 ^−6^) had strikingly different *lacZ* MFs in AA-exposed mice, possibly because AA (as an acid) can quickly become solid and form deposits in the forestomach, leading to a higher local concentration. This observation further demonstrated that AA induces mutations in a tissue-specific manner.

### *Hprt* gene

The *Hprt* gene, located on the X-chromosome and encoding hypoxanthine-guanine phosphoribosyltransferase (HPRT), has been widely used as a reporter gene for somatic mutations *in vivo*. More than three decades ago, the mutagenicity of AA on the *Hprt* locus was observed in rats using a granuloma pouch assay, in which AA (undefined composition of AAI and AAII) was directly injected to a subcutaneous air pouch.^[71]^ Furthermore, a single oral dose of 45 or 90 mg/kg AA resulted in a significant induction in *Hprt* MF although only two rats were tested in the treated group. Using a more sophisticated technique, we reported that AA exposure increased MFs in the spleen T-cell *Hprt* gene.^[61]^ A statistically significant increase was seen even in the low-dose group in which animals were treated with 2.75 mg/kg bw AA for 28 consecutive days. The *Hprt* MF in the high-dose group (11 mg/kg bw) was 55-fold higher than that in controls, confirming that AA is a potent mutagen in rats. The duration of exposure also plays an important role in determining AA’s mutagenicity. Given the same dose (11 mg/kg bw), the T-cell *Hprt* MF following a 28-day treatment was about 5-fold greater than that in a 3-day treatment.

### *Pig-a* gene

The *Pig-a* assay, which uses the phosphatidylinositol glycan, Class A gene as a reporter of mutation, is a recommended *in vivo* tool by the International Workshop on Genotoxicity Testing (IWGT) for the evaluation of chemical-induced mutagenicity.^[72]^ Two separate experiments were performed on male F344 rats to examine the mutagenicity of AA using the *Pig-a* assay.^[61]^ In the first experiment, animals were treated with a daily dose of AA mixtures (0, 2.75, 5.5, and 11 mg/kg bw) for 28 days, while the second experiment used higher doses (0, 11, 22, and 30 mg/kg bw) for a shorter time (3 days). On the 4^th^ day after the treatments, the relative RET fraction, indicating bone marrow toxicity, was reduced by AA in a dose-dependent manner in both experiments. The %RET frequencies recovered to the levels comparable to the controls by day 14 for all the dose groups. The *Pig-a* MFs, as measured by a number of CD59-deficient total red blood cells (RBC^CD59−^) and RET (RET^CD59−^), were evaluated on days 1, 15, and 29 in both experiments. The results showed the *Pig-a* MFs were significantly increased by AA in a dose- and time-dependent manner. The increases for RBC^CD59−^ and RET^CD59−^ MFs were up to 137- and 128-fold compared to the vehicle controls, respectively, supporting a mutagenic MOA for AA carcinogenesis.

Recently, another study examined the *Pig-a* MF in male Sprague-Dawley rats treated with a single dose of either 0, 15, 30, and 60 mg/kg bw AA (1:1 mixture of AAI and AAII) through oral gavage.^[73]^ Peripheral blood was collected 7, 14, and 28 days after dosing. They found that AA treatment increased the *Pig-a* gene MF in a time-dependent manner. The elevated MF of RBC^CD59−^ was identified at day 28 but not in earlier time points. The RET^CD59−^ MF appeared to be a more sensitive marker, as statistically significant increases were found at days 7, 14, and 28 for the high-dose groups. It should be noted that the RETs were quickly regenerated after AA induced acute erythropoietic toxicity. Therefore, it is expected that the induction of MFs in the RET^CD59−^ occurs earlier than in the RBC^CD59−^ in peripheral blood.

### *Ras* proto-oncogenes

*Ras* gene family members, including three isoforms, i.e. H-*Ras*, K-*Ras*, and N-*Ras*, are key genes regulating cell growth and division. Their association with various types of cancers has been studied extensively in the past decades.^[74]^ *Ras* mutation often occurs at codons 12, 13, or 61. A single mutation in the *Ras* gene can activate the oncogene. For example, a mutation in the H-*Ras* leads to aberrant functions and is correlated with the development of urinary tract cancer.^[75]^ The correlation between AA exposure and *Ras* mutation *in vivo* was initially identified in the 1990s. To investigate the mechanism of AAI-induced carcinogenicity, Schmeiser *et al.* treated male Wistar rats with 10 mg/kg bw AAI five times per week orally for 3 months.^[76]^ A total of 35 tumors from various tissues were developed and analyzed. They found that AAI activated the *Ras* genes and A: T to T: A transversion at codon 61 was the predominant mutation type. Furthermore, this H-*Ras* mutation was identified in 100% of squamous cell carcinomas of the forestomach, 93% of forestomach tumors, and 100% of ear duct tumors. Concordantly, in mice, many A: T to T: A H-*Ras* transversions were observed in neoplastic tissue sections, while such a mutation was absent in adjacent normal tissues.^[77]^ Given that dA-AAI is the major and persistent DNA adduct formed upon AAI exposure, this A: T to T: A transversion is not unexpected and consistent with a mutagenic MOA for AA-induced tumor induction.

**Figure 5:**
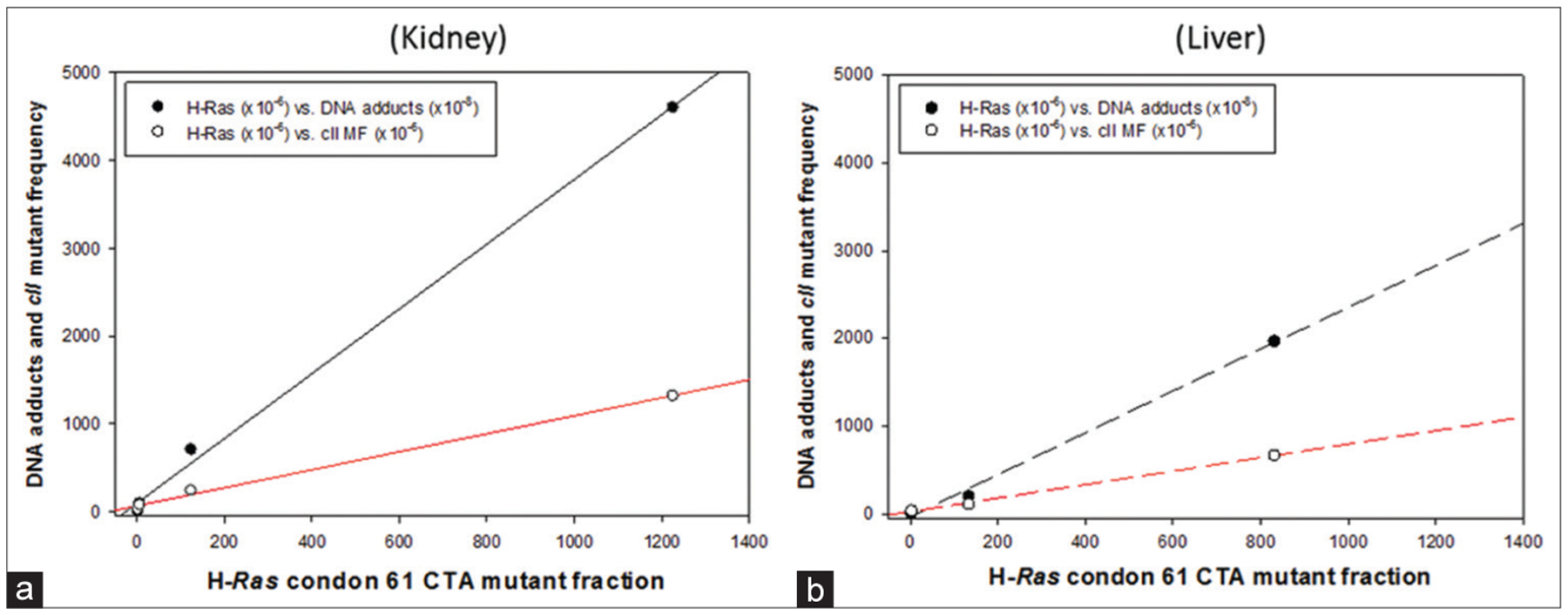
Correlations between H‑*Ras* codon 61 CTA mutant fraction and DNA adducts (black lines) or *cII* mutant frequencies (red lines) in the Kidney (a) and liver (b) from control and 10 mg/kg aristolochic acid‑treated rats. H‑*Ras* data are from Wang *et al*.,^[38]^ while total DNA adduct levels and *cII* mutant frequencies are from Figures 2 and 3. Open dot: H-*Ras* mutant fraction vs. *cII* mutant frequency, Close dot: H-*Ras* mutant fraction vs. DNA adducts

DNA samples from Big Blue transgenic rats were used to investigate *Ras* mutation as a potential key event in the MOA for AA’s carcinogenesis. An unique allele-specific competitive blocker–polymerase chain reaction (ACB-PCR) technique was used to detect CAA to CTA (H-*Ras*) and GGT to GAT (K-*Ras*) mutations.^[38]^ H-*Ras* mutant fraction at codon 61 increased in a dose-dependent manner in both liver and kidney of the AA-treated rats. Significant correlations between the H-*Ras* mutant fraction and the total burden of AA-DNA adducts and the *cII* gene MFs were identified [Figure 5], suggesting that H-*Ras* mutations play an important role in AA’s tumorigenesis. On the other hand, no correlation was observed between K-*Ras* mutation and AA-DNA adduct formation in either the kidney or the liver. It is argued that while at lower doses AA may amplify the spontaneous K-*Ras* mutation in target organs, the overt cytotoxicity at higher doses may trigger apoptosis in cells with preexisting K-*Ras* mutation, which can, in turn, influence the results of the mutation assays.

### *p53* tumor suppressor gene

Multiple human studies demonstrated that AA induces mutations in the tumor suppressor gene *p53* through the signature A: T to T: A transversion, which contributes to the overexpression of mutated p53 protein and carcinoma formation in the urinary tract.^[78,79]^ A mouse model knocked-in with human *p53* (Hupki) was used to evaluate the role of p53 in the carcinogenicity of AA.^[80]^ Primary Hupki embryonic fibroblast cells were exposed to 100 μM AAI for 48 h and passaged for 8–10 weeks. Base substitution mutations were identified in 50% of the established embryonic fibroblast cell lines, with A to T transversion as the major mutation type. Particularly, this A to T mutation in codon 139 of the p53 DNA-binding domain matched with that identified in the urothelial tumor in a patient exposed to AA.^[81]^ In addition, lacking/inhibiting *p53* appears to attenuate AAN in mice. Tubular epithelial cell necrosis and apoptosis in the renal cortex is one of the major pathological features in chronic AAN. Tubular cell death was significantly decreased in *p53* knockout mice, compared to wild-type controls treated with the same dose of AA.^[82]^ Pretreatment of pifithrin-α, a p53 inhibitor, also ameliorated acute AAN in C57BL/6 mice. Phosphorylation of p53 increases the stability of the protein and thus enhances its activity. On activation, p53 can trigger cascades of downstream signaling pathways that can cause cell cycle arrest and induce apoptotic cell death. Indeed, p53 was found to be activated by phosphorylation in mice treated with 10 mg/kg AA for 3 days.^[82]^ It has been well-documented that AA exposure results in an accumulation of DNA adducts in the kidney, initiating genotoxic stress. Previous evidence suggests that such genotoxic stress can potentially contribute to the posttranscriptional activation of p53.^[83]^ Therefore, posttranscriptional modification of the tumor suppressor genes through genotoxicity may serve as another indirect mechanism for AA’s carcinogenic effects.

## Aristolochic Acid-Induced Toxicogenomic Changes

### Gene expression profiles in aristolochic acid-treated rodents

Gene expression profiles generated through microarray or next-generation sequencing (NGS) technologies allow a better understanding of molecular mechanisms in chemical carcinogenesis at the transcriptomic level. To this end, our research group compared the gene expression profiles in the kidney (target tissue) and liver (nontarget tissue) of Big Blue transgenic rats exposed to 10 mg/kg AA (containing 40% AAI and 56% AAII) for 3 months^[39]^ because rats treated with AA at this dose developed tumors in the kidney.^[9]^ Principal component analysis (PCA) and hierarchical cluster analysis (HCA) clearly visualized the different gene expression profiles for the tissues and AA treatment. First, the liver samples (both the control and AA-treated samples) were well separated from all kidney samples, indicating a large difference between the two tissues. Second, kidney or liver samples from the rats exposed to AA were grouped together and clearly separated from their control group, suggesting that AA exposure can be identified based on gene expression profiles. Using the criteria of *P* < 0.01 and fold change >2, a total of 1174 and 838 differentially expressed genes (DEGs) were identified in the kidney and liver, respectively [Table 1]. In addition, AA-treated kidney had more DEGs with higher fold changes when compared to the liver, suggesting that AA treatments resulted in greater gene expression alterations in the kidney. Functional analysis of these DEGs indicated that many more DEGs in kidney were altered in cancer-related pathways, including cell growth and proliferation, defense response, immune response, apoptosis, and tumor morphology. We reported that tumor suppressor genes such as *Inhba* were increased as a potential defense mechanism against AA-associated genotoxicity in the kidney. In addition, a literature shows the activation of tumor suppressor gene *p53* by AA in mice.^[72]^ More recently, Arlt *et al.* examined the gene expression profiles of the kidney and liver in mice knocked in with human *P53*.^[29]^ They identified a significant enrichment of nuclear factor-kappa B, p53, and cell cycle pathways in the kidney of AAI-treated animals. An increase in mRNA and protein levels of the *cMyc* oncogene was also observed. Collectively, gene expression profiles indicate that AA significantly altered multiple carcinogenic pathways in rodent kidney and the differential alterations between the target and nontarget tissues suggest tissue-specific mechanisms of AA-induced toxicity and carcinogenicity.

In a later study, five different microarray platforms were compared using samples from AA-treated rat kidney and the results from the different platforms consistently showed carcinogenesis-related changes in the biological functions and pathways.^[84]^ When NGS technologies became available, the gene expression profiles generated by NGS with those from microarray technologies using the same samples were compared.^[85]^ A total of 1,416 DEGs were commonly identified by both methods, with 98.2% (1390) DEGs exhibiting changes in the same direction. In the biological process category, there were 62 gene ontology terms commonly enriched by two platforms, with more DEGs identified with NGS than microarray. These results suggest that the gene expression profiles can elucidate molecular mechanisms and provide an improved understanding of AA-induced toxicity.

### MicroRNA expression profiles in aristolochic acid-treated rodents

MicroRNAs (miRNAs) are a class of small (~22 nucleotides in length) noncoding RNAs that regulate gene expression at the posttranscription level. Growing evidence indicates that miRNAs are extensively involved in chemical carcinogenesis and miRNA expression profiles are informative for identifying the genotoxicity and carcinogenicity of chemicals.^[86]^ To determine if miRNAs could be used as tissue-specific biomarkers for AA-induced genotoxicity, miRNA expression profiles in the liver and kidney of rats treated with 10 mg/kg AA were examined using a miRNA microarray analysis (LC Sciences, Houston, TX, USA) that contains 359 rat miRNAs.^[40]^

A total of 247 miRNAs, expressed in at least one tissue sample, were used for the PCA and HCA analyses. The liver samples were clearly separated from the kidney samples, indicating that miRNA expression profiles in the two tissues are different, with the separation of the control and AA-treated kidney tissues and nonseparation between the control and AA-treated liver tissues. Of 247 detectable miRNAs, 10 mg/kg AA treatment resulted in 19 differentially expressed miRNAs in the kidney using criteria of *P* < 0.05 and fold change >2, whereas only one miRNA was changed in the liver [Table 1]. Among the 19 miRNAs differently expressed after AA treatment in the kidney, 11 of them were downregulated (all were <3-fold) and 8 were upregulated (five miRNAs, i.e. miR-21, miR-34a, miR-10b, miR-182, and miR-30e, were >3-fold). These 19 miRNAs altered in the kidney were all associated with carcinogenesis. For example, both miR-21 and miR-34a showed higher fold changes (>9) [Table 1] in AA-treated kidney. miR-34a was the only miRNA upregulated (5.2-fold) in AA-treated liver. Further studies indicated that miR-34a was upregulated dose dependently both in the kidney and liver, and the expression levels of miR-34a positively correlated with the DNA adduct levels and *cII* MF.^[40]^ These data suggest that miRNA expression profiles can distinguish AA-induced genotoxic and carcinogenic insult in the target kidney from nontarget liver, and therefore, miRNA expression profile may be able to supplement genotoxicity endpoints.

Recently, deep-sequencing technologies (also called high-throughput sequencing or NGS) have become the powerful technology of choice. Our deep-sequencing data for global miRNA and mRNA expression showed that AA treatment resulted in 63 miRNAs and 6794 mRNAs altered significantly in rat kidney.^[87]^ This miRNA deep-sequencing analysis identified 417 detectable miRNAs, which was higher than those identified through miRNA microarray assay (247 detectable miRNAs).^[40]^ There were 13 miRNAs (including miR-34a) upregulated >10-fold and only one (i. e., miR-383) with >10-fold downregulation. Functional annotation indicated that the top diseases/functions related to these miRNAs were “cancer, organismal injury, and abnormalities,” suggesting that dysregulated miRNA expression plays an important role in AA-induced carcinogenesis in rat kidney.^[87]^

### Proteomic expression profiles in aristolochic acid-treated rodents

Proteomics is the large-scale study of proteins, and a major effort, related to proteomics, has been biomarker development. Toxicoproteomics can be used to elucidate toxicological responses at the protein level, i.e., the analysis of protein expression, modifications, protein–protein/toxicant interactions, and protein activities.^[88]^ A metabonomic study of AA-induced nephrotoxicity in Wistar rats found that certain metabolic pathways, such as homocysteine formation and folate cycle, were significantly accelerated.^[89]^ Using trypsin-catalyzed ^16^O/^18^O labeling in conjunction with two-dimensional liquid chromatography separation and tandem mass spectrometry, proteomes of rat kidney were quantitatively analyzed in our proteomic study. Greater than 9,000 unique peptide sequences were identified, with 800 proteins that were significantly altered by AA treatment.^[87]^ For most cancer-related targets, protein expression showed expression patterns similar to that observed from miRNA expression. For example, after a 12-week AA treatment, cancer-related targets that were associated with downregulated (12) and upregulated (54) miRNAs had their proteins expressed in the same direction (downregulation 75% [9/12] and upregulation 83% [45/54]). On the other hand, the correlation of expression between protein expression and mRNA was relatively weak, suggesting that more factors in addition to miRNAs are involved in posttranscriptional regulation.

## Benchmark Dose Modeling of Aristolochic Acid-Induced Genotoxicity Data

Benchmark dose (BMD) modeling has been introduced to the genotoxicity field for quantitative analysis of *in vitro* and *in vivo* dose–response relationships.^[90,91]^ The BMD is a dose used for estimating a predetermined change of adverse response over control (the benchmark response) and is preferred by the Working Group on Quantitative Approaches to Genetic Toxicology Risk Assessment of the IWGT over other point-of-departure (PoD) metrics, such as the no observed genotoxic effect level and the breakpoint dose.^[92]^ The two-sided lower (BMDL) and upper (BMDU) bounds of BMD 95% confidence intervals can be generated and used for quantitative comparisons of test article-induced genotoxicity across multiple endpoints. The BMDLs are the preferred PoDs by toxicologists.^[93]^ Currently, two software packages are generally acceptable for calculating the BMD values using mathematically modeled dose–response curves, i.e., BMD Software (BMDS) and PROAST, which were developed by the U. S. Environmental Protection Agency and the Netherlands’ National Institute for Public Health and the Environment, respectively.

We performed BMD analyses on two *in vivo* genotoxicity endpoints in three organs. Specifically, the continuous data model in BMDS (version 2.7, released in 2017; https://www.epa.gov/bmds/benchmark-dose-software-bmds-version-27-materials) was used to analyze *cII* MF dose–response curves for the kidney, liver, and spleen^[35–37]^ and H-*Ras* mutations in the kidney and liver^[38]^ of Big Blue transgenic rats gavaged with AA at 0.1, 1, and 10 mg/kg for 12 weeks. The BMDs producing a 10% (BMD_10_), 50% (BMD_50_), and 100% (2-fold; BMD_100_) increase over the background frequencies were calculated using the five models (i.e., exponential, Hill, linear, polynomial, and power) in BMDS used for modeling continuous data. The model that produced the lowest Akaike’s information criterion along with absolute values of scaled residuals ≤ ±2 was chosen as the best model for calculating the BMDs and their BMDLs and BMDUs.^[94]^ Accordingly, the Hill model was used for analyzing the liver and kidney *cII* MF data, the linear model was selected for the spleen *cII* MFs and liver H-*Ras* mutation data, and the polynomial model was employed for the kidney H-*Ras* mutation data.

**Figure 6:**
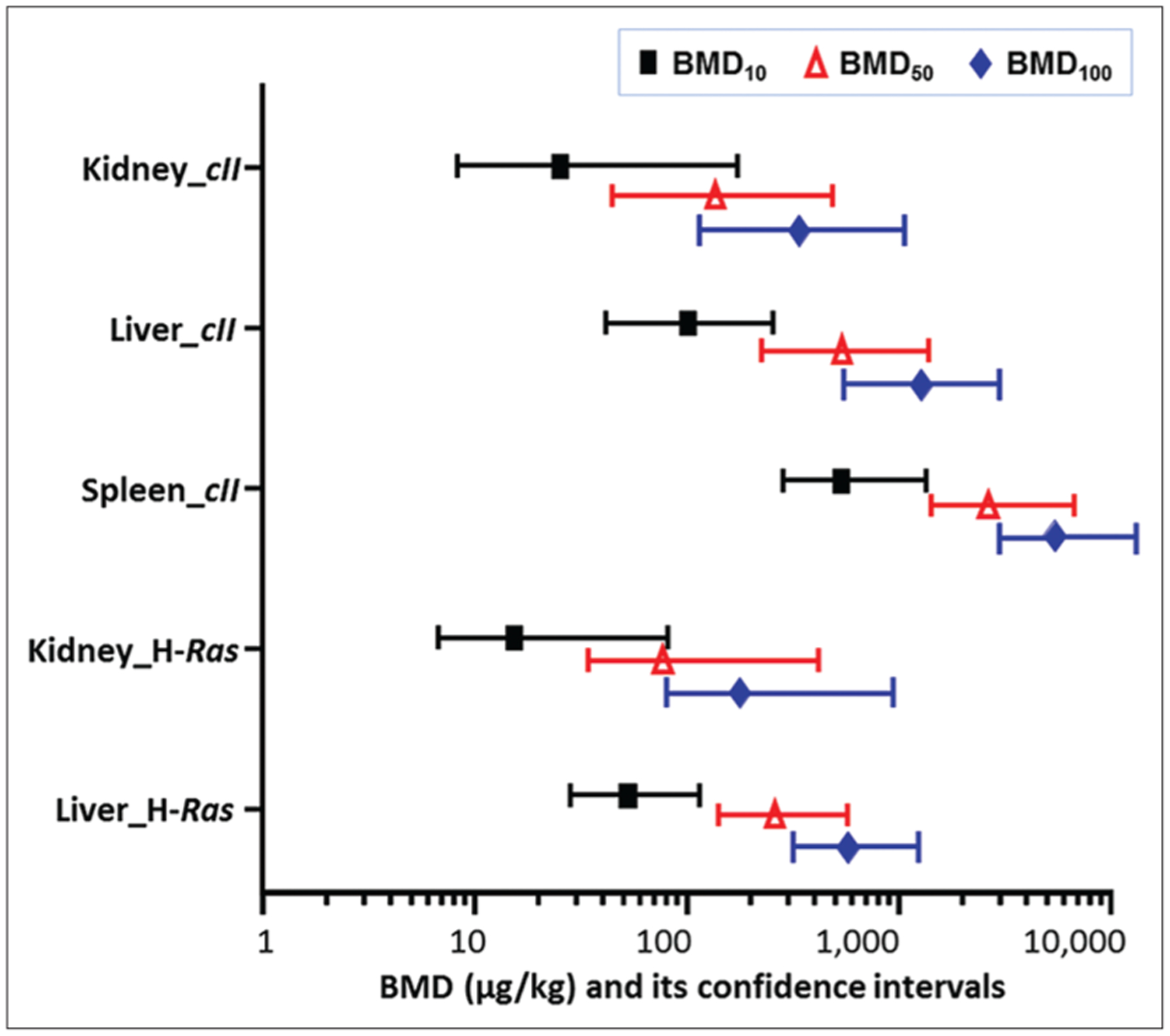
Comparison of benchmark dose values for the *cII* mutant frequencies and H-*Ras* mutant fractions induced by aristolochic acid. The benchmark dose values producing a 10%, 50%, and 100% increase over the background level were calculated using Benchmark dose software package. The bars indicate the calculated lower and upper 95% confidence interval (BMDL and BMDU) of each value. Black bar, BMD_10_; red bar, BMD_50_; and blue bar, BMD_100_

The BMD_10_ and its BMDL_10_ have historically been applied for quantitative risk assessment, and such a 10% increase may not be seen at some endpoints.^[95]^ In agreement with the fact that AA induced the highest *cII* and H-*Ras* mutation responses in the kidney, the BMDL_10_s (the lower the more mutagenic) for both endpoints were the lowest in the kidney, followed by liver and spleen [Table 2 and Figure 6]. The BMDL_10_s for inducing *cII* mutations in kidney, liver, and spleen were 8, 41, and 286 μg/kg/day, respectively, i.e., a daily dose of 8 μg/kg AA will increase *cII* MF 10% above the background level in the kidney after 3 months of treatment. For the H-*Ras* mutations, the BMDL_10_ for kidney (7 μg/kg/day) was about 4-fold lower than that of the liver (28 μg/kg/day); this is to say that a daily dose of 7 μg/kg AA results in a 10% increase in H-*Ras* oncogene mutation over the control level in the kidney after 3-month treatment. The two BMDL_10_s calculated from the dose–response curves of *cII* MF and H-*Ras* mutation in the kidney were very close [Table 2]. Using the body surface area normalization method,^[96]^ these BMDL_10_ of 7–8 μg/kg/day for both *cII* MF and H-*Ras* mutation in the rat kidney can be converted to a human equivalent dose of 1.1–1.3 μg/kg/day which equates to 66–78 μg/day for a 60 kg person. Therefore, a 60 kg person who consumes AA at 66–78 μg/day for 3 months may potentially have 10% more mutations that may have increased risk for cancer development. With modifying factors (uncertainty or safety factors), the permitted daily exposure defined as a pharmaceutically acceptable intake can also be calculated^[97]^ and could be much lower than the BMDL_10_ for genotoxicity. It has been reported that the BMDL_10_ for AA-related end-stage renal disease is 420 μg cumulative AA exposure.^[8]^ However, in a worst-case scenario, exposure to >130 mg of AA can be achieved in 1 week.^[24]^ Considering the Belgian AAN cases, this 130 mg cumulative chronic consumption dose is associated with a higher risk of cancer; its conversion to a daily dose in rats (supposing 12-week treatment, 159 μg/kg/day) is about 2-fold higher than the BMDL_100_ for *cII* MF and H-*Ras* mutation in the kidney [Figure 6]. The more ingestion or inadvertent exposure to AA-containing herbal products, the higher the risk of human diseases, including cancer.

## Concluding Remarks

Chemical carcinogenesis can be attributed to a genotoxic or nongenotoxic MOA. *In vivo* models, particularly rodents, have long been used to predict the carcinogenic potential of genotoxic compounds.^[98]^ Since the genotoxic MOA was likely to be human relevant, the results from *in vivo* genotoxic tests (i.e., the *in vivo* comet, micronucleus, and reporter gene mutation assays) can be informative in cancer risk assessment. There was little research on AA-induced genotoxicity until the unfortunate incident in Belgium in the early 1990s, where a group of young women ingested a mixture of “slimming drugs” accidently containing *A. fangchi*. These AA exposures led to the formation of DNA adducts in multiple tissues in this group^[50,51]^ and also induced the signature mutation (A: T to T: A) that contributed to the formation of urothelial cancer. Our interest in investigating AA-induced genotoxicity was inspired by these reports. During the last two decades, we have determined AA-induced DNA adducts, *cII* mutant frequencies, and H-*Ras* mutation fraction in the kidney, liver, and spleen of rats exposed to AA for 12 weeks [Figures 2, 5 and Table 1]. Since many new technologies have been developed and toxicogenomics has become an important subdiscipline in the field of toxicology,^[99]^ we have also performed several toxicogenomic studies to better understand the biological processes driving AA-induced genotoxicity and carcinogenesis [Table 1]. In addition, we applied BMD modeling to find the lowest dose of AA that could induce mutations and discovered that the BMD lower confidence limit (BMDL_10_) for AA-induced mutations was about 7–8 μg/kg/day in rat kidneys [Table 2].

Recently, a molecular epidemiologic study indicated that AA exposure significantly contributed to upper urinary tract urothelial carcinoma in 151 patients in Taiwan.^[27]^ In addition, the new results using whole-genome sequencing techniques have linked AA exposure to some cancers in Asia in additional tissues, such as bladder and liver.^[100,101]^ Although the findings for hepatocellular carcinoma were based on a small sample size^[102]^ and the A: T to T: A mutational signature was not observed after AA exposure alone,^[103]^ these data are consistent with AA’s genotoxic MOA. The findings for liver cancers have renewed concerns about public health risk which is rapidly become a public debate in China.^[104]^ In the early 2000s, the China Food and Drug Administration (CFDA) abolished the use of a few AA-containing medicinal materials, and in October of 2017 (just after publication of the AA liver cancer finding), the CFDA listed some Chinese medicines that contained AA on its official website for warning purposes.^[104]^ It is worth noting that the market for traditional Chinese medicines is huge and Chinese herbs have also been exported as raw materials to over 175 countries, for example, Japan, South Korea, Germany, the Netherlands, and the USA.^[105]^ Most herbal products are formulated in the USA, but the raw plant materials for herbal products are largely imported from China.^[7]^

Dietary exposure to AA and its derivatives is associated with the development of primary and secondary cancers in humans. Because AA-containing plants and products are not totally excluded in many countries and commonly used as dietary supplement products, AA-induced nephrotoxicity and carcinogenicity is now recognized as a worldwide health issue. While educating consumers and increasing global public awareness are very important for primary prevention, more investigation will help build the body of evidence and elucidate the molecular and cellular mechanisms of AA-induced toxicity, genotoxicity, and carcinogenicity. The generation, analysis, integration, and interpretation of the massively increased data obtained from *in vitro*, *in vivo*, and human studies are critical steps for better understanding AA-induced toxicity, providing new insights into the causes of human cancer, facilitating the development of biomarker screening for at-risk populations, and promoting public health.

## Figures and Tables

**Table 1:** Fold changes of genotoxic endpoints and toxicogenomic changes after aristolochic acid treatments

10 mg/kg AA treatment (for 12 weeks)	Group	Fold change (AA vs. control)				Our study
		Kidney	Liver	Spleen	Testis	
DNA adducts*	dA-AAI	99	75	53	−	[35,37]
	dG-AAI	47	88	11	−	
	dA-AAII	41	73	47	−	
	Total adducts	49	79	47	−	
*cII* MF	All *cII* gene	45.5	23.8	8.1	1.1	[35–37,40]
	A:T→T:A^#^	25	11	6	–	
H-*Ras* mutant fraction	Median value	412	280	–	–	[38]
	Mean value	130	356	–	–	
Deferentially expressed genes (*P*<0.01)	2–5	1050	788	–	–	[39]
	5–10	95	34	–	–	
	>10	29	16	–	–	
	Sum	1174	838			
miRNA microarray assay (*P*<0.05)	2–5	16	0	–	–	[40]
	5–10	3	1	–	–	
	>10	0	0	–	–	
	Sum	19	1			
miRNA deep sequencing (*P*<0.05)	2–5	25	–	–	–	[87]
	5–10	12	–	–	–	
	>10	14	–	–	–	
	Sum	51				

* Fold changes for DNA adducts were calculated by the values in 10 mg/kg group divided by those in 0.1 mg/kg group due to no DNA adducts (“0”) in the control group,

# Fold changes for A:T→T:A (as a signature mutation) were calculated based on the percentage in its mutation spectrum. −: Not determined, MF: Mutant frequency, miRNA: MicroRNA, AA: Aristolochic acid, dA-AA: 7-(deoxyadenosin-*N*^6^-yl)-aristolactam

**Table 2:** Comparison of the lower limits of benchmark doses (BMDLs) producing a 10%, 50%, and 100% increase over the background level (BMDL_10–100_) for the five dose responses

BMDL (*μ*g/kg)	*cII* MF			H-*Ras* mumtation	
	Kidney	Liver	Spleen	Kidney	Liver
BMDL_10_*	8	41	286	7	28
BMDL_50_	45	225	1430	34	141
BMDL_100_^#^	98	500	2859	68	282

* The BMDL_10_ has historically been used for the quantitative risk assessment,

# The BMDL_100_ (i.e., a two-fold increase) has commonly used for the assessment of genotoxicity data. MF: Mutant frequency, BMDL: The lower limit of benchmark dose (BMD)
